# Evaluating the Microstructure and Bioaccessibility of Bioactive Compounds and Antioxidant Activity After the Dehydration of Red Cabbage

**DOI:** 10.3390/foods14111932

**Published:** 2025-05-29

**Authors:** Nicol Mejias, Antonio Vega-Galvez, Alexis Pasten, Elsa Uribe, Ana Andrés, Sara Muñoz-Pina, Kateryna Khvostenko, Purificación García-Segovia

**Affiliations:** 1 Food Engineering Department, Universidad de La Serena, Av. Raúl Bitrán 1305, La Serena 1700000, Chile; susana.mejias@userena.cl (N.M.); afpasten@userena.cl (A.P.); muribe@userena.cl (E.U.); 2 Instituto Universitario de Ingeniería de Alimentos-FoodUPV, Universitat Politècnica de València, Camino de Vera s/n, 46022 Valencia, Spain; aandres@tal.upv.es (A.A.); samuopi@upvnet.upv.es (S.M.-P.); kkhvost@upvnet.upv.es (K.K.); pugarse@tal.upv.es (P.G.-S.)

**Keywords:** brassica vegetables, drying kinetics, glucosinolates, simulated digestion

## Abstract

This study aims to examine the effects of various drying methods, namely convective drying (CD), vacuum drying (VD), infrared drying (IRD), low-temperature vacuum drying (LTVD), and freeze-drying (FD), on the microstructure and in vitro bioaccessibility of red cabbage bioactive compounds and antioxidant activity. Total polyphenol content (TPC), total flavonoid content (TFC), total anthocyanin content (TAC), total glucosinolate content (TGC), individual phenolic profile, and antioxidant potential (DPPH, ABTS, FRAP) were examined experimentally in original and digested samples. Overall, SEM images of dried red cabbage showed more porous structures and minimal parenchyma deformation, enhancing bioactive compound bioaccessibility. Specifically, the bioaccessibility of TPC in IRD-dried samples was remarkably higher than that of FD- and LTVD-dried samples, although the latter two proved more TAC and TGC bioaccessible, respectively. Notably, hydroxycinnamic acids, such as ferulic and p-coumaric acids, markedly increased after digestion, especially in FD-dried samples. In vitro digestion revealed that CD-dried samples showed stronger DPPH and FRAP radical scavenging, whereas FD-dried samples had significantly higher ABTS scavenging activity. These findings suggest that LTVD and FD are the most suitable drying methods for red cabbage to enhance relevant bioactive compounds and, to some extent, antioxidant activity after digestion.

## 1. Introduction

Red cabbage (*Brassica oleracea* var. capitata f. rubra) is a crop recognized for its characteristic purple-colored heads and its substantial amounts of bioactive compounds (vitamins, dietary fibers, flavonoids, cyanidin-based anthocyanins, hydroxycinnamic acid, and glucosinolates) that promote good health [1,2,3]. Among the flavonoid class, red cabbage anthocyanins have been shown to function as antioxidants, as they may help reduce reactive oxygen species [4,5]. Together with anthocyanins, glucosinolates are associated with inhibiting tumor formation when they are hydrolyzed to isothiocyanates [6]. However, the antioxidant capacity and content of such compounds can be affected by drying processing, the extent of which depends on the drying condition and its duration [7]. For instance, IR drying utilizes radiation to dehydrate the sample without the need for a heat transfer medium, whereas CD depends on the movement of hot air for moisture removal [8]. Nevertheless, both techniques may induce the degradation of heat-sensitive compounds and structural modifications [9]. VD is employed to preserve heat-sensitive compounds [1]. Nevertheless, the rapid evaporation of water under reduced pressure can lead to condensation on the cooler surfaces of the chamber, which may not be efficiently removed by the vacuum system [10]. LTVD and FD are both low-temperature dehydration methods conducted under reduced pressure, effectively preserving cell structure integrity and bioactive compounds [7,11,12]. Nonetheless, their high energy consumption and prolonged processing durations result in elevated operational costs [1,13]. Previously, we demonstrated that VD, FD, and LTVD present advantages over conventional CD and IRD methods in retaining anthocyanins, glucosinolates, polyphenols, and flavonoids, and the DPPH radical scavenging of red cabbage due to the restriction of oxygen and reduced temperatures [12]. Similarly, Yue et al. [1] demonstrated that the restriction of oxygen and the short drying time achieved through microwave vacuum drying (MVD) performed well in terms of TFC, TPC, TAC, and antioxidant ability, as well as the microstructure of purple cabbage. Xu et al. [14] combined MVD with CD or VD, developing a promising technology for drying cabbages, which exhibited better preservation of nutrient composition and antioxidant activity. Li et al. [15] reported that microwave freeze-drying (MFD) and FD were able to preserve the viable probiotics, bioactive compounds, and antioxidant activities of fermented napa cabbage more effectively than CD. Nonetheless, it is worth noting that a high retention of bioactive components after drying does not necessarily translate to equivalent biological activity in the human body [16]. The biological activity is closely linked to the bioaccessibility of the bioactive components. Bioaccessibility means the proportion of a compound that is released from the food matrix during gastrointestinal digestion and becomes available for absorption in the small intestine [17]. However, the stability and extractability of bioactive compounds during digestion are highly influenced by the surrounding conditions, which ultimately impact their concentration and activity in the bioaccessible fraction [18]. These factors are modulated by the food’s macro- and microstructure, its chemical composition, and other matrix-related characteristics, all of which can undergo significant alterations during processing [19]. Thus, the drying process may affect both the abundance of bioactive compounds and the microstructure of the food matrix (i.e., the disruption of cell walls and membranes); consequently, this could affect the bioaccessibility of bioactive compounds by increasing the release of them from the cell [20]. Santos et al. [3] demonstrated that different powdered matrices obtained from dried purple cabbage by different methods significantly influenced bioactive compound release during gastric digestion. They reported that spray drying resulted in the higher bioaccessibility of total phenolic content (TPC) and total anthocyanins (TA) than spouted bed drying, FD, and CD. However, there have been few studies interested in the relationship between drying method-induced changes in the red cabbage microstructure and their bioactive compounds in in vitro bioaccessibility. Therefore, in this study, the relationship between the microstructure, the bioaccessibility of bioactive compounds, and the antioxidant activity of red cabbage subjected to convective drying (CD), vacuum drying (VD), infrared drying (IRD), low-temperature vacuum drying (LTVD), and freeze-drying (FD) was assessed.

## 2. Materials and Methods

### 2.1. Chemicals and Reagents

All reagents used in this study were of analytical grade and obtained from Sigma-Aldrich (St. Louis, MO, USA). Distilled water, methanol, Folin-Ciocalteu reagent, sodium carbonate (Na_2_CO_3_), Gallic acid standard, sodium nitrite (NaNO_2_), Aluminium chloride (AlCl_3_), sodium hydroxide (NaOH), Cathequin standard, potassium chloride (KCl), sodium acetate (CH_3_COONa), ferric chloride hexahydrate (FeCl_3_×6H_2_O), sodium tetrachloropaladate II (Na_2_PdCl_4_), hydrochloric acid (HCl), potassium persulfate (K_2_S_2_O_8_), sinigrin standard, 2,2-diphenyl-1-picrylhydrazyl (DPPH), 6-hydroxy-2,5,7,8-tetramethylchromo-2-carboxylic acid (Trolox), 2,4,6-tripyridyl-s-triazine (TPTZ), and 2,2′-azino-bis(3-ethylbenzthiazoline-6-sulfonic acid (ABTS). All digestive reagents and enzymes required for the bioaccessibility assay were purchased and prepared according to the standardized protocol described by Minekus et al. [21].

### 2.2. Preparation of Study Materials, the Drying Procedure, and Microstructure Analysis

**Figure 1:**
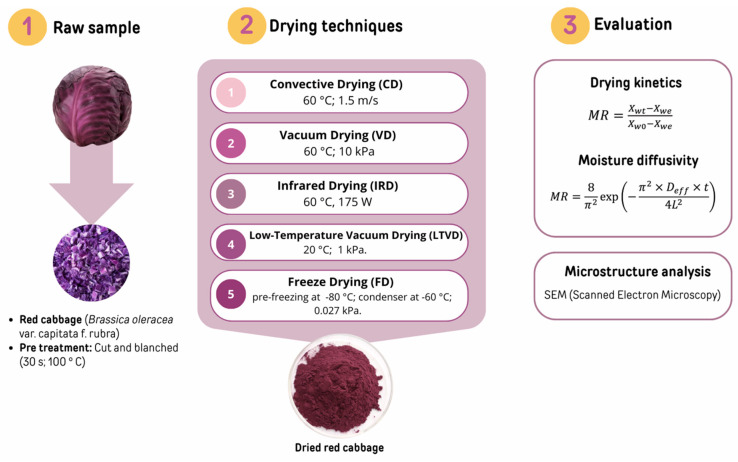
Graphic representation of the preparation of red cabbage and drying procedure.

Fresh red cabbage (*Brassica oleracea* var. capitata f. rubra) was obtained from a commercial supplier in La Serena, Chile. The cabbage heads were manually cut into uniform segments of approximately 1 cm in length using a domestic vegetable slicer. The pieces were subjected to a blanching process consisting of immersion in boiling water for 30 s, immediately followed by rapid cooling in an ice water bath to partially denature the epithiospecific protein. After cooling, excess surface moisture was removed using a vegetable centrifuge. The blanched and drained material was then homogenized and divided into five equal portions, each of which was subjected to a different drying method. Specific conditions for each drying treatment are detailed in our previous work [12] and summarized in Figure 1.

The study established the conditions for temperature, pressure, radiant power, and air velocity to ensure effective moisture removal while preserving the bioactive compounds and health-promoting properties of red cabbage. By adopting these validated parameters, the present work ensured methodological consistency and enabled a direct comparison of the results.

The drying kinetics evaluation can be described in terms of the moisture ratio (MR), the variation in moisture content (X_wt_) at any processing time (t), the initial moisture content (X_w0_), and the equilibrium moisture (X_we_) (Figure 1). All were expressed as grams of water per gram of the dry sample. The diffusional process in drying cabbage can be expressed by the term effective moisture diffusion coefficient (Dₑ) in m^2^/s, where L is half the thickness of the dried materials, assuming an infinite slab geometry and constant moisture diffusivity.

Microstructure analysis was conducted using scanning electron microscopy (SEM) as described by Vega-Galvez et al. [22]. Rehydrated samples were mounted on a cryo-holder and rapidly frozen using slush nitrogen (a mixture of solid and liquid N_2_). The chamber temperature was maintained at –135 °C, and frozen water was sublimated at –90 °C under high vacuum conditions (2 × 10⁻^6^ mbar). The sample surface was then coated with a platinum layer for 30 s at a current of 5 mA. Micrographs were obtained at 500× magnification. The electron accelerating voltage was set at 3.00 kV, optimized to balance resolution and sample charging. Observations were conducted using a field emission scanning electron microscope (FESEM) (Zeiss Ultra 55, Carl Zeiss AG, Oberkochen, Germany). Multiple fields of view were examined per replicate, and representative micrographs were selected based on consistent structural features observed across all samples, ensuring reproducibility of the microstructural observations.

### 2.3. Procedure for Extracting Bioactive Compounds from Red Cabbage

#### 2.3.1. Extracts Obtained from Dried Red Cabbage

Bioactive compounds were extracted from each dried red cabbage sample by a conventional solvent extraction procedure. Four grams of dried sample was transferred into conic tubes and mixed with 15 mL of aqueous methanol 80% and extracted by agitation at 250 rpm for 1 h at room temperature (Orbital Shaker, OS-20, BOECO, Hamburg, Germany). After the agitation time, tubes were centrifuged (Eppendorf 5804 R, Hamburg, Germany) at 5000 rpm for 20 min and at 4 °C, and then the supernatants were collected. This process was repeated up to three times to ensure a complete extraction. This extract was used as the control for comparison with the extracts obtained after in vitro digestion.

#### 2.3.2. Extracts Obtained During Simulated In Vitro Gastrointestinal Digestion

**Figure 2:**
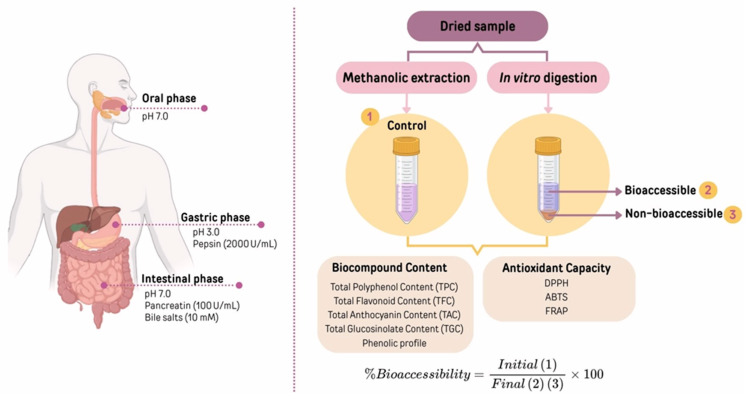
Graphic representation of the simulated gastrointestinal digestion and extraction procedure.

The simulated gastrointestinal digestion was performed following the procedure described by Minekus et al. [21]. This consisted of a three-step process designed to mimic digestion in the mouth (oral phase), stomach (gastric phase), and small intestine (intestinal phase). A representation of the simulated gastrointestinal digestion procedure is shown in Figure 2.

Three simulated digestion fluids were prepared and reserved: simulated salivary fluid (SSF), simulated gastric fluid (SGF), and simulated intestinal fluid (SIF). Each fluid was formulated using 1.25× concentrated electrolyte stock solutions to compensate for subsequent dilution upon enzyme and water addition, ensuring physiologically relevant final ion concentrations. The SSF included key electrolytes such as KCl, KH_2_PO_4_, NaHCO_3_, and MgCl_2_·6H_2_O, and was adjusted to pH 7.0. SGF contained NaCl, KCl, NaHCO_3_, KH_2_PO_4_, and MgCl_2_·6H_2_O, and was acidified to pH 3.0 using 1 M HCl. For SIF, the electrolyte mixture was supplemented with NaCl, NaHCO_3_, KH_2_PO_4_, KCl, MgCl_2_·6H_2_O, and CaCl_2_ to achieve a final pH of 7.0. Enzymes such as porcine pepsin (2000 U/mL in SGF), pancreatin (adjusted to 100 U/mL trypsin activity in the final mixture), and bile salts (10 mM) were added to their respective digestion phases. CaCl_2_ was incorporated in all phases to reach final concentrations of 0.75, 0.075, and 0.3 mM for SSF, SGF, and SIF, respectively. All digestion fluids were prepared fresh using ultrapure water and handled under clean laboratory conditions, including the use of sterile plasticware and autoclaved glassware, and were pre-warmed to 37 °C prior to use. Briefly, 1 g of each dried red cabbage sample was mixed with 5 mL of SSF, and the pH was adjusted to 7.0. The mixture was homogenized by gentle vortexing for 10 s and then incubated in an Intelli-Mixer RM-2 (Elmi Ltd., Riga, Latvia) at 25 rpm inside a thermostatic chamber maintained at 37 °C (J.P. Selecta SA, Barcelona, Spain). After 2 min of incubation (oral phase), 10 mL of SGF containing porcine pepsin (2000 U/mL) was added. The pH was adjusted to 3.0 using 1 M HCl, and the mixture was incubated under the same conditions for 2 h (gastric phase). Subsequently, 10 mL of SIF, supplemented with pancreatin (adjusted to 100 U/mL trypsin activity) and bile salts (10 mM), was added. The pH was readjusted to 7.0 with 1 M NaOH, and the mixture was incubated for an additional 2 h at 37 °C (intestinal phase). Throughout the digestion process, samples were gently but thoroughly agitated to ensure homogeneous contact between enzymes and substrates, and the pH was monitored and adjusted if necessary. To halt enzymatic activity after the intestinal phase, Bowman–Birk Inhibitor from soybean (Sigma-Aldrich, St. Louis, MO, USA) was added to the digestion mixture, followed by immediate cooling on ice. Finally, the digests were centrifuged, and 1 mL aliquots of the supernatant were collected using sterile techniques into sterile Eppendorf tubes and stored at −80 °C for subsequent analysis. A blank digestion control was prepared using ultrapure water handled under the same clean laboratory conditions instead of the sample.

#### 2.3.3. Extracts Obtained from Digesta

Whole digesta samples (Figure 2) were subjected to the conventional solvent extraction procedure described in the Section 2.3.1. This extract represents the non-bioaccessible fraction. Water was also used as a blank.

### 2.4. Bioactive Compound Quantification of Extracts

#### 2.4.1. Quantification of Polyphenol Content

The total polyphenol content (TPC) was estimated for each extract, i.e., control, bioaccessible, and non-bioaccessible fractions, according to the Folin-Ciocalteu method [23]. At the beginning, 20 µL of extract was mixed with 1.58 mL of deionized water and 100 µL of Folin-Ciocalteu reagent. After 3 min, 300 µL of Na_2_CO_3_ (20% *w*/*v*) was added and incubated in the dark for 60 min, and then absorbance was read at 765 nm (UV/VIS, Beckman Coulter du 730, Brea, CA, USA). Gallic acid was used as a standard for a calibration curve from 0 to 500 mg/L (y = 0.002x − 0.09; R^2^ = 0.9982). The results were expressed as mg of gallic acid equivalent (GAE) (mg GAE/g dry matter [d.m.]).

#### 2.4.2. Quantification of Flavonoid Content

The total flavonoid content (TFC) was conducted based on the work of Muñoz-Pina et al. [24] for each extract (i.e., control, bioaccessible, and non-bioaccessible fractions). Overall, 100 µL of reconstituted red cabbage extracts was mixed with 1.4 mL of deionized water followed by addition of 75 µL of NaNO_2_ (5%). After 6 min, 150 µL of AlCl_3_ was included and let set for 5 min, then 500 µL of NaOH 1 M was added. Absorbance was read and quantification was made with cathequin (+) as a standard (y = −0.0159x − 0.00138; 0.9995). Results were expressed as mg cathequin equivalent (CAE)/g d.m.

#### 2.4.3. Quantification of Anthocyanin Content

The total anthocyanin content (TAC) was determined by the pH differential method based on the work of Ahmadiani et al. [25] for each extract (i.e., control, bioaccessible, and non-bioaccessible fractions). Two buffers were used to dilute the extracts: potassium chloride (pH 1.0; 0.25 M) and sodium acetate (pH 4.0; 0.4 M). The absorbance of each buffer-extract mix was read by a spectrophotometer (UV/VIS, Beckman Coulter du 730, California, USA) at 510 and 700 nm. TAC was determined by the following equations:

(1) $$∆A=\left(A_{510}-A_{700}\right){pH}_{1.0}-\left(A_{510}-A_{700}\right){pH}_{4.5}$$

(2) $$TAC=\frac{∆A\times M_{W}\times DF\times 1000}{Ɛ\times 1}$$

where, *M_W_* is the molecular weight of cyanidin-3-glucoside (cya3glu) equivalent to 449 g/mol; *DF* is the dilution factor; Ɛ is the molar extinction coefficient of cya3glu (26,900). The results are expressed in terms of mg cya3glu equivalents/g d.m.

#### 2.4.4. Quantification of Glucosinolates Content

The total glucosinolate content (TGC) was determined by a spectrophotometric assay [26] for each extract, i.e., control, bioaccessible, and non-bioaccessible fractions. Sixty microliters of extract were mixed with a solution (2 mM) of sodium tetrachloropaladate II (Na_2_PdCl_4_), previously prepared with 170 µL of concentrated HCl and 100 mL double distilled water. The mixture was incubated in the dark for 30 min at room temperature. Then, absorbance was read at 450 nm (UV/VIS, Beckman Coulter du 730, Garden Grove, CA, USA), in which high absorbances are related to high TGC. A sinigrin calibration curve was made (1.25–10 mM). The results were expressed as µmol sinigrin equivalent (SE)/g d.m.

#### 2.4.5. Quantification of Individual Phenolic Compounds

A phenolic profile was carried out using an HPLC (Agilent 1200 Series Rapid Resolution, Agilent, Palo Alto, CA, USA) attached to a diode array detector (Agilent 1260 Infinity II, Agilent, Palo Alto, CA, USA) according to Tanleque-Alberto et al. [27]. The extracts, i.e., control, bioaccessible, and non-bioaccessible fractions, were filtered with a PTFE filter with a 0.45 μm pore (Scharlab, Barcelona, Spain). Phenolic compounds were separated using a Brisa-LC 5 μm C18 column (250 × 4.6 mm) (Teknokroma, Barcelona, Spain). The separation was carried out with a mobile phase consisting of 1% formic acid (phase A) and acetonitrile (phase B), following a programmed gradient elution: 90% A at 0 min, 85% A at 3 min, 60% A at 18 min (held until 24 min), 34% A at 27 min, 50% A at 28 min, 30% A at 33 min, 10% A at 40 min, and returning to 90% A at 43 min (held until 45 min). The total chromatographic runtime was 45 min. The column temperature was maintained at 30 °C, with a flow rate of 0.5 mL/min and an injection volume of 10 μL.

The chromatographic column was maintained at 30 °C, operating at a flow rate of 0.5 mL/min, with an injection volume of 10 μL. The identification of unknown compounds was conducted by comparing their retention times with those of reference standards. Detection wavelengths were assigned as follows: 250 nm for vanillic acid, 260 nm for 4-hydroxybenzoic acid, rutin, quercetin 3-glucoside, and quercitrin; 280 nm for gallic acid, epicatechin, quercetin, and trans-cinnamic acid; 290 nm for naringenin; 320 nm for 4-O-caffeoylquinic acid, caffeic acid, p-coumaric acid, sinapic acid, ferulic acid, and apigenin-7-glucoside; and 380 nm for kaempferol. Data acquisition and processing were carried out using Agilent MassHunter Workstation software (version B.09.00). The results are expressed as μg/g dry basis, calculated from a standard calibration curve.

### 2.5. Antioxidant Potential of Extracts

#### 2.5.1. DPPH Radical Scavenging Assay

The DPPH assay was determined based on the methodology by Brand-Williams et al. [28] with some modification. Previously, a DPPH stock solution was prepared by diluting 24 mg of DPPH in 100 mL of methanol and then reserved. A stock solution was mixed with methanol until reaching an absorbance of 1.10 ± 0.02 in a 1 cm cuvette read at 515 nm. Ten microliters of extracts, i.e., control, bioaccessible, and non-bioaccessible fractions, were taken and mixed with 2850 µL of the previous DPPH solution, allowing it to react for 30 min in the dark. The synthetic antioxidant Trolox (6-hydroxy-2,5,7,8-tetramethylchromo-2-carboxylic acid) was used as the standard in a calibration curve from 25 to 800 µM Trolox (y = −0.0009x + 1.0853; R^2^= 0.9913). The results were expressed as µmol Trolox equivalents (TE)/g d.m.

#### 2.5.2. Ferric-Reducing Antioxidant Power Assay

The antioxidant FRAP assay was carried out according to Thaipong et al. [29], in which a working solution was prepared with 25 mL of 300 mM buffer acetate pH 3.6, 2.5 mL TPTZ (2, 4, 6-tripyridyl-s-triazine), 10 mM in 0.04 M HCl, and 2.5 mL FeCl_3_ × 6H_2_O 20 mM. Before the assay, the working solution was warmed at 37 °C, and then 285 µL was mixed in a spectrophotometer cuvette with 25 µL of red cabbage extract and 125 µL of distilled water and incubated for 30 min in the dark. Absorbance was read to determine the colored product (ferrous tripyridyltriazine complex) at 593 nm. Trolox was used as a standard to make a calibration curve from 0 to 100 µg/mL (y = 0.0103x + 0.0053; R^2^= 0.9967). The results were expressed as µmol TE/g d.m.

#### 2.5.3. ABTS Radical Cation Scavenging Capacity Assay

The antioxidant ABTS assay was performed based on the work of Thaipong et al. [29], in which a stock solution was prepared with 5 mL ABTS 7.4 mM in methanol and 10 mL 2.6 mM potassium persulfate (K_2_S_2_O_8_) aqueous solution. The stock solution was kept in the dark for 12 h, and then 3.4 mL was mixed with 120 mL of methanol to reach an absorbance of 1.10 ± 0.02 at a 734 nm wavelength. 5 µL of extract was diluted in 145 µL of water and mixed with 2850 µL of adjusted ABTSand kept in the dark for 2 h allowing it to react. Absorbance was read at 734 nm, and a calibration curve for ABTS was plotted with Trolox concentrations from 0 to 200 µg/mL (y = −0.0052 + 1.029; R^2^ = 0.9937), and results were expressed as µmol TE/g d.m.

### 2.6. Statistical Analysis

The results were expressed as means ± standard deviation and statistically analyzed using the RStudio software (V. 1.4.1717). The means were compared by an analysis of variance (ANOVA) and Tukey’s test to estimate the significance among the main effects at the 5% probability level. Heat map plots displaying Pearson correlations between variables were generated using Python version 3.9.4 (Python Software Foundation, Beaverton, OR, USA).

## 3. Results and Discussion

### 3.1. Drying Kinetics

**Figure 3:**
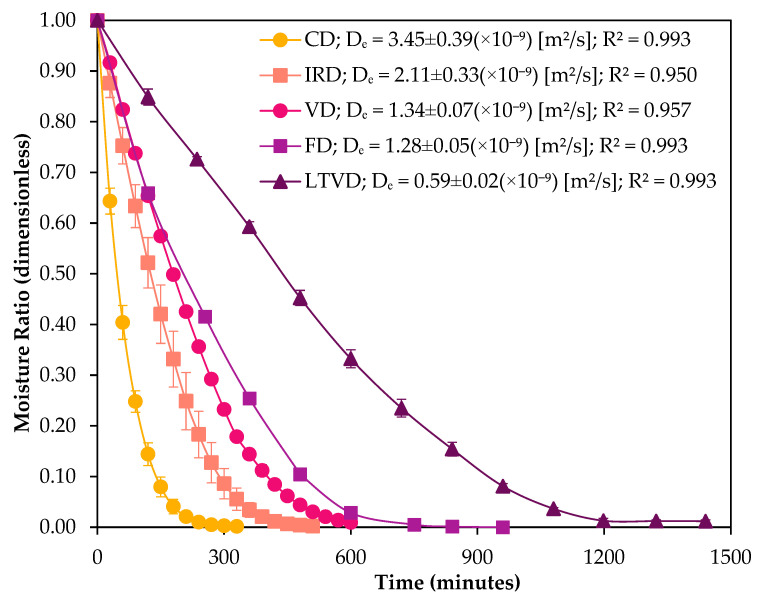
Changes in the moisture ratio (MR) of red cabbage dried with different methods. Values are the means of triplicate analyses (*n* = 3), and error bars are the standard deviation. CD: convective drying; VD: vacuum drying; IRD: infrared drying; LTVD: low-temperature vacuum drying; FD: freeze-drying.

The drying time and moisture ratio (MR) curves for dried red cabbage under the different methods are presented in Figure 3.

The graph revealed a significant difference in drying time among the various drying methods. The shortest drying times were observed for CD and IRD (330 and 510 min, respectively), whereas the longest drying times were recorded for VD, FD, and LTVD methods (600, 960, and 1440 min, respectively). These results suggest that the hot air flow in CD was a key factor in reducing drying time and MR [30]. It is because heat transfer occurs layer by layer via hot air, promoting greater diffusion of water trapped within the hemicellulosic cellular structure of cabbage [31]. Although the radiant energy of IRD can penetrate the inner layers of the material, its energy attenuation with depth and moisture diffusion were lower than those of hot air [32]. Slower drying speed observed in VD, FD, and LTVD is due to the limited amount of air inside the chamber caused by the vacuum environment. In the absence of airflow, water removal occurs primarily through pressure gradients that transport water molecules away from the product [33]. In FD, water vapor is removed by sublimation and captured as ice in a cold condenser [34]. In VD and LTVD, the vacuum pump continuously extracts water vapor from the drying chamber. However, the water rate vaporization will depend on the material temperature and vacuum pressure degree [33]. This behavior is supported by the effective diffusivity values (Figure 3) calculated from the Fick’s diffusion equation. Consistently, the dried sample obtained by CD has the highest Dₑ value (3.45 × 10^−9^ m^2^/s) among the drying methods, followed by the IRD-dried sample (2.11 × 10^−9^ m^2^/s). In vacuum systems, the temperature used in VD enhances Dₑ (1.34 × 10^−9^ m^2^/s), allowing faster drying compared to FD (1.28 × 10^−9^ m^2^/s) and LTVD (0.59 × 10^−9^ m^2^/s). These variations in Dₑ reflect differences in drying efficiency across methods [33]. Nonetheless, the Dₑ values obtained in this study were found to be of the same order of magnitude as those reported for cabbage in the literature [22,31]. The differences in Dₑ values among the drying methods can also be explained by the internal microstructure of the dried samples, which will be discussed in the next section.

### 3.2. Microstructure Analysis

**Figure 4:**
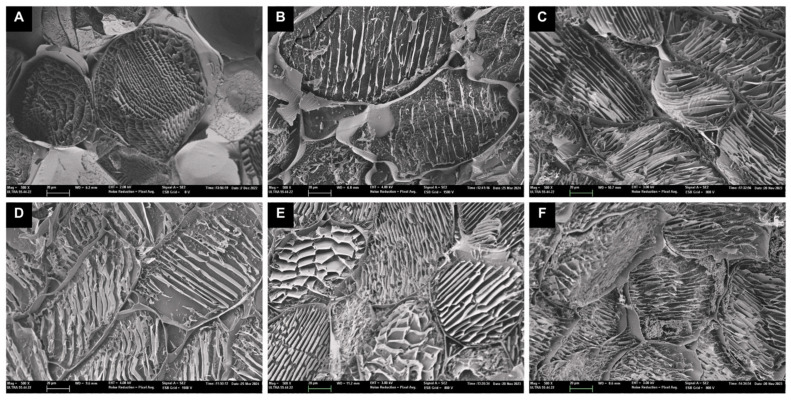
Changes in the microstructure of red cabbage dried with different methods. (**A**) fresh sample; (**B**) CD: convective drying; (**C**) VD: vacuum drying; (**D**) IRD: infrared drying; (**E**) LTVD: low-temperature vacuum drying and (**F**) FD: freeze-drying.

To better understand the drying mechanism, a microstructure analysis is essential, as it provides qualitative information on the morphological alterations occurring in the material after drying [35]. SEM micrographs of fresh and dried red cabbage obtained from five different drying methods (magnified at 500×) are presented in Figure 4.

As shown in Figure 4A, the cells of fresh red cabbage remained intact, and the polygonal-shaped parenchyma structure was well preserved. This was consistent with the microstructure of fresh cabbage observed by Pongmalai et al. [36]. In general, dehydration caused different degrees of parenchyma cell wall deformation across the drying methods. CD appears to cause the most deformation to the parenchyma cells and to induce partial collapse of the tissue structure, with visibly deformed and compressed parallel cell lines (Figure 4B), likely resulting from convective airflow and mechanical stress during drying. This structural compaction reduces overall porosity but may generate longitudinal channels that promote moisture migration [37], which is consistent with the highest Dₑ value reported in Figure 3. The IRD-dried sample (Figure 4D) exhibited significant microstructure improvements over that dried by CD. This could result partly from the radiant energy of IRD, which penetrates the sample surface and generates localized internal heating. This may facilitate moisture migration from the inner matrix to the surface [38], resulting in partial disruption of cell walls and the formation of internal channels, which could create additional pathways for moisture diffusion [39]. The VD-treated sample (Figure 4C) showed moderate structural damage, with partial collapse and reduced porosity compared to LTVD (Figure 4E) or FD (Figure 4F); however, the higher temperature applied in VD likely enhanced internal vapor pressure and accelerated diffusion [40], resulting in a higher Dₑ than LTVD and FD (Figure 3). In contrast, samples treated by FD and LTVD, although preserving highly porous and open microstructures with a uniform honeycomb-like pattern and minimal parenchyma deformation [1,14], exhibited lower Dₑ values due to the longer time needed to generate an adequate vapor pressure gradient under low-temperature conditions [41]. Therefore, the more compact structure observed in VD-dried samples compared to LTVD and FD may be attributed to the combined effect of vacuum and higher processing temperatures, which could intensify the pressure gradient between internal and external layers of the tissue, promoting structural shrinkage and collapse in the absence of counteracting pressure forces [14].

In conclusion, both structural characteristics and drying conditions (mainly temperature and pressure) interact to determine the overall diffusivity and efficiency of the drying process. It is worth noting that this study did not include a quantitative analysis of microstructural parameters such as porosity, pore size distribution, or cell deformation degree. However, future research will incorporate image analysis techniques to enable a more detailed and objective comparison of microstructural characteristics across different drying treatments.

### 3.3. Drying Changes Bioactive Components in Red Cabbage

Previously, we investigated the effects of the studied drying methods on the bioactive compounds and antioxidant properties of red cabbage [12]. In a general context, we demonstrated that VD, FD, and LTVD would propose advantages over conventional CD and IRD methods in retaining anthocyanins, glucosinolates, polyphenols, flavonoids, and DPPH radical scavenging due to the restriction of oxygen and reduced temperatures. A similar behavior was found in the current study. Other authors have also reported a high retention of bioactive compounds and antioxidant ability in different cabbage cultivars as a result of shorter drying times, low temperatures, and limited oxygen availability in MVD, MFD, and FD processes under appropriate conditions [1,14,15,42]. Nonetheless, it is worthy to note that a high retention of bioactive components after drying does not necessarily translate to equivalent biological activity in the human body [16]. The biological activity is closely linked to the bioaccessibility of the bioactive components, which is the main goal of this study. The effect of the drying method on the bioaccessibility of biocompounds and antioxidant properties in red cabbage after in vitro digestion is discussed in detail below.

### 3.4. Drying Influences the Bioaccessibility of Bioactive Components in Red Cabbage After In Vitro Digestion

As already stated, in addition to quantifying TPC, TFC, TAC, and TGC in red cabbage samples subjected to different drying methods, we also evaluated the bioaccessibility of these compounds after in vitro digestion. The results are presented in Table 1.

Overall, the initial values of TPC of dried red cabbage obtained from different methods ranged from 10.53 to 12.64 mg GAE/g d.m., which is consistent with the range reported in the literature for dried red cabbage [1,5]. The two-way ANOVA revealed significant effects of both the drying method and digestion status on the TPC (*p* < 0.05), as well as a significant interaction between the two factors. After digestion, there was a significant (*p* < 0.05) increase in the TPC of all samples. This increase was expected and may be attributed to the alkaline pH of the intestine, along with the action of pancreatic enzymes and bile salts, which facilitate the release and biotransformation of phenolic compounds, thereby enhancing their bioaccessibility and bioactive potential following simulated digestion [43]. As shown in Table 1, the highest initial value of TPC is shown in CD-dried samples (12.64 mg GAE/g d.m.), followed by FD-dried samples (11.39 mg GAE/g d.m.). The lowest value is in IRD-dried samples (10.53 mg GAE/g d.m.). Interestingly, digestion resulted in the most pronounced increase in TPC in the IRD-dried sample, reporting a bioaccessibility index of 296%. In contrast, CD- and FD-dried samples exhibited more moderate increases of 173% and 138%, respectively. This indicates that a high TPC content in the dried sample prior to digestion does not necessarily translate into greater bioaccessibility. Thus, the statistically significant interaction suggests that the impact of digestion on phenolic compound release is strongly dependent on the drying method employed, likely due to differences in matrix structure and the chemical stability of phenolic compounds. Infrared radiation, in particular, can induce localized cell wall disruption and partial structural breakdown through rapid surface heating, which may enhance the release of polyphenols, flavonoids, and other phenolic compounds [44,45]. Although IRD did not produce a globally porous and homogeneous microstructure, as observed in FD (Figure 4F) and LTVD (Figure 4E), the cellular fractures and surface openings present in IRD-treated samples (Figure 4D) likely contributed to the higher extractability and bioaccessibility of phenolics. In contrast, despite the superior preservation of porous structures in FD and LTVD samples, the lower temperatures employed in these methods may not have been sufficient to fully inhibit the activity of oxidative enzymes. As a result, these enzymes may have remained active even after drying and during the initial phase of digestion, potentially contributing to the degradation of polyphenol compounds through oxidative enzymatic activity [18,46]. Nonetheless, the lowest TPC bioaccessibility was observed in samples processed with VD. This might be due to the fact that VD-dried samples exhibit a compact and dense cellular structure (Figure 4C), which hinders the release of polyphenol compounds during digestion, thereby decreasing their bioaccessibility in comparison to the other dried samples studied.

On the other hand, initial values of the TFC of dried cabbage ranged from 3.78 to 4.49 mg CAE/g d.m. (Table 1), which is consistent with the range reported in previous studies [1,4,42,47]. As observed, the TFC was increased after digestion to a certain extent. However, the bioaccessibility of flavonoids tends to be much lower than polyphenols. In fact, the total recovery of flavonoids only reached a value of 123% for IRD-dried samples and 113% for CD-dried samples after digestion. Both drying methods appear to cause greater deformation of the parenchyma cells in cabbage, which could disrupt glycosidic bonds and release free flavonoids under the acidic and enzymatic conditions of the gastric environment, making them to some extent more bioaccessible and quantifiable [48]. A two-way ANOVA revealed a significant interaction between drying method and digestive status (*p* < 0.05), indicating that the effect of digestion on TFC depends on the drying technique used. In particular, CD and IRD showed statistically significant increases in TFC after digestion (*p* < 0.05), while no significant differences were observed for VD, LTVD, and FD (*p* > 0.05), as confirmed by pairwise comparisons. This may be due to the interaction of flavonoids with proteins and dietary fiber in cabbage, which provides a physical barrier against acidic gastric conditions, thereby limiting their solubilization and release [49]. However, this interaction allows flavonoid retention in the non-digestible fraction [50], with values ranging from 0.47 to 0.86 mg CAE/g d.m (Table 1).

Among flavonoids, anthocyanins are the most abundant in red cabbage. As shown in Table 1, the initial values ranged from 2.62 to 5.65 mg Cya3glu/g d.m. The highest TAC was observed in FD-dried cabbage, followed by LTVD (5.17 mg Cya3glu/g d.m.), while the lowest values were found in IRD-dried samples. The restriction of oxygen and the use of low temperatures in LTVD and FD methods may explain the highest TAC values. On one hand, the vacuum environment inhibits anthocyanin degradation through oxidation [1]; whereas, on the other hand, low temperatures prevent their thermal cracking [51]. In addition, both methods produce more porous structures, which enhance solvent penetration into the plant matrix and thereby improve the extraction efficiency of anthocyanins [18]. When the bioaccessibilities of anthocyanins were evaluated, there was a significant (*p* < 0.05) decrease in TAC values for CD, VD, and LTVD samples with respect to initial values, suggesting that anthocyanin degradation occurred during simulated digestion. This was associated with their instability during the pH transition from the acidic medium in the stomach to the alkaline condition in the small intestine [20,52]. On the contrary, the FD process increased the bioaccessibility of anthocyanins. It is probable that the dense, honeycomb-like structure may physically entrap anthocyanins, limiting their exposure to digestive enzymes and pH variations during digestion, thereby enhancing bioaccessibility of anthocyanins [51]. Although the TAC in IRD-dried cabbage improves after digestion, it remains lower than in other dried samples (Table 1). We speculate that the recovery of polyphenols in IRD samples involves the formation of linkages with the vegetable matrix during digestion, which may protect more labile anthocyanins from degradation [52]. Finally, it can be observed that the non-digestible fraction of anthocyanins ranged from 0.47 to 0.86 mg Cya3glu/g d.m. The formation of these precipitated and non-digestible compounds results from the interaction of anthocyanin-based complexes with proteins and bile salts [53].

Overall, limited information is available in the scientific literature on the relationship between drying method-induced changes in the red cabbage microstructure and their bioactive compound bioaccessibility, limiting data comparison. Santos et al. [3] demonstrated that different powdered matrices obtained from dried purple cabbage by different methods significantly influenced bioactive compound release during gastric digestion. They reported that spray drying resulted in higher bio-accessibility of TPC and TAC than other drying methods.

Regarding glucosinolates, the initial TGC ranged from 78.53 to 94.78 µmol SE/g d.m. for dried cabbage using different methods (Table 1). These values are higher than those reported in the literature [7,54]. However, it is important to note that TGC was determined spectrophotometrically using sinigrin as the standard in our study, which typically results in higher values compared to the sum of individual glucosinolates quantified by chromatographic techniques. According to the ANOVA followed by Tukey’s post hoc test (*p* < 0.05), LTVD-dried samples exhibited the highest TGC, which was significantly higher than that of IRD-dried samples. No significant differences were observed among CD, VD, and FD treatments, which presented intermediate values. These results suggest that LTVD may better preserve thermolabile glucosinolates under low-temperature and vacuum conditions, whereas IRD might promote greater degradation, potentially due to direct infrared radiation and localized heating effects [12]. According to the two-way ANOVA, both the drying method and the digestion process significantly influenced the TGC (*p* < 0.05). In all cases, the bioaccessible fraction exhibited significantly higher glucosinolate levels than the corresponding non-digested controls, showing an increase between 1.18-fold and 1.55-fold. This finding is consistent with the literature, which describes glucosinolates as molecules containing a sulfate group and a thioglucose moiety, conferring them high water solubility and, consequently, high bioaccessibility in the intestinal lumen [6,55]. However, this observation contrasts with previous studies. For instance, Fernández-León et al. [56] reported a 69% loss in total glucosinolate content in Savoy cabbage after an in vitro digestion assay, whereas García-Pérez et al. [57] reported only a 30.9% loss in fresh red cabbage. Abellán et al. [58] even reported complete degradation of glucosinolates in red cabbage sprouts after digestion. In all these studies, the degradation of glucosinolates occurred because myrosinase was not inactivated prior to in vitro digestion. This implies that the myrosinase–glucosinolate interaction mechanism occurred during the digestion simulation to be converted into isothiocyanates [59]. In our study, although the short blanching time (30 s) followed by cooling applied to fresh samples before drying may have been insufficient to fully inactivate myrosinase [7], the additional thermal and physical stress associated with the drying processes likely contributed to a further reduction in its enzymatic activity. This could explain the observed increase in bioaccessible glucosinolates, as partial inactivation of myrosinase may have prevented significant enzymatic hydrolysis, allowing some intact glucosinolates to reach the intestinal phase [60]. However, further investigation of residual myrosinase activity and the identification of individual glucosinolates are necessary to validate and strengthen the interpretation of our findings.

### 3.5. Drying Influences the Bioaccessibility of Individual Phenolic Compounds in Red Cabbage After In Vitro Digestion

The phenolic compound profiles of the in vitro gastrointestinal digested extracts were compared with non-digested extracts (control) by HPLC-DAD (Table 2).

Six different compounds, including gallic acid, 4-O-caffeoylquinic acid, caffeic acid, vanillic acid, ferulic acid, and p-coumaric acid, were identified. These compounds have also been identified by other authors in red cabbage [61,62,63]. Vanillic acid was the most abundant in the non-digested extracts (control), especially in the FD-dried (0.83 mg/g d.m.) and LTVD-dried samples (0.60 mg/g d.m.). The CD-dried, VD-dried, and IRD-dried samples exhibited vanillic acid concentrations of 0.40 mg/g d.m., 0.29 mg/g d.m., and 0.09 mg/g d.m., respectively. All other compounds were present in concentrations ranging from 0.02 to 0.18 mg/g d.m. (Table 2). Phenolic acids are known to be heat-sensitive and may degrade at 60 °C, which is the temperature applied during the CD, VD, and IRD processes. Although this degradation may reduce the amount of phenolic compounds, thermal processing can also break down certain complex polyphenols into small phenolic compounds, which may exhibit improved bioaccessibility [64]. In fact, both ferulic and p-coumaric acid showed a notable increase (*p* < 0.05) in concentrations after digestion in all samples. Specifically, the first compound increased up to 15-fold, and the second up to 12-fold, in the FD-dried samples. Gallic acid was absent in the non-digested extracts obtained from LTVD- and FD-dried red cabbage, but appeared after digestion, with LTVD showing the highest levels. It has been reported that gallic acid is generated through the degradation of cyanidin-3-O-glucoside, resulting in smaller monomeric and dimeric compounds with potentially higher bioaccessibility [65]. Nevertheless, it has also been reported that anthocyanins may degrade via the chalcone pathway into smaller phenolic compounds during simulated gastrointestinal digestion, particularly in response to pH variations. Among these degradation products, phenolic acids such as ferulic acid and p-coumaric acid have been identified, which could explain the increase of such phenolic acids after simulated digestion [66,67,68]. Furthermore, FD and LTVD have been noted to control the microstructure of red cabbage, which facilitates the release of phenolic compounds from their matrix, thereby increasing the bioaccessibility and/or extractability [69]. In contrast, caffeic acid did not show significant changes overall. However, vanillic acid concentration showed a decreasing trend after simulated digestion, decreasing up to 82.8% with respect to initial values. Likewise, 4-O-caffeoylquinic acid was found only in the non-digested extracts of all dried cabbages, suggesting both compounds are degraded during in vitro digestion [68]. It is possible that enzymes present during the simulated digestion may hydrolyze the ester linkage in 4-O-caffeoylquinic acid, resulting in the degradation of the parent compound. Meanwhile, vanillic acid could be susceptible to decarboxylation or enzymatic oxidation under gastrointestinal conditions [70].

### 3.6. Drying Influences the Bioaccessibility of Antioxidant Propierties in Red Cabbage After In Vitro Digestion

**Figure 5:**
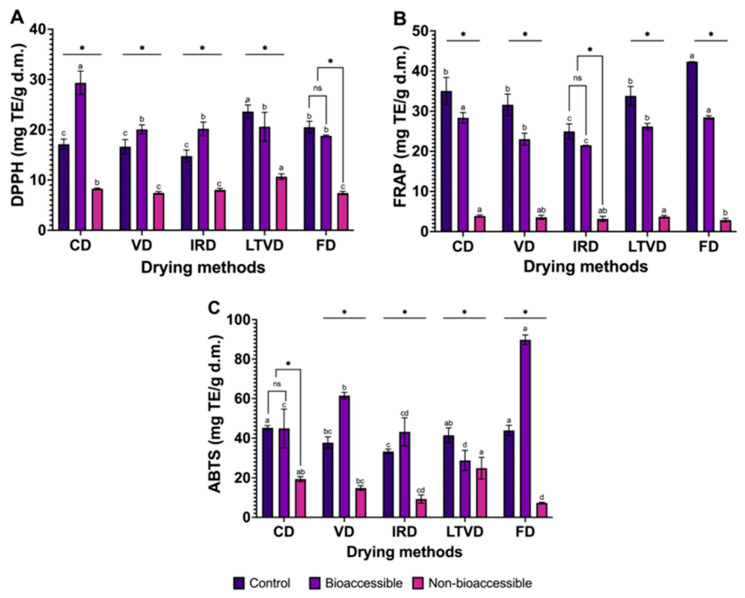
Changes in the antioxidant potential of dried red cabbage using different methods during in vitro gastrointestinal digestion evaluated by (**A**) DPPH assay, (**B**) FRAP assay, and (**C**) ABTS assay. CD: Convective Drying; VD: Vacuum Drying; IRD: Infrared Drying; LTVD: Low-Temperature Vacuum Drying; FD: Freeze-Drying. Values are expressed as mean ± standard deviation (*n* = 3). Different letters within bars indicate significant differences (*p* < 0.05) among drying methods. Asterisks (*) indicate significant differences (*p* < 0.05) among fractions (control, bioaccessible, and non-bioaccessible fraction) within each drying method.

The antioxidant potential of non-digested (control) and in vitro gastrointestinal digested extracts obtained from dried red cabbage by different methods was measured using three assays with two accepted mechanisms of antioxidant activity: radical scavenging activity (DPPH and ABTS assays) and electron donation (FRAP assay) (Figure 5).

Based on the statistical analysis, the antioxidant potential of non-digested (control) extracts varied significantly depending on the drying method and the assay employed. In the DPPH assay (Figure 5A), LTVD-dried samples exhibited the significantly highest value (23.64 mg TE/g d.m.). In the FRAP assay (Figure 5B), FD-dried samples showed the highest activity (42.33 mg TE/g d.m.). For the ABTS assay (Figure 5C), the highest antioxidant activities were observed in CD, FD, and LTVD samples (44.64, 43.90, and 41.43 mg TE/g d.m., respectively). The different antioxidant potential levels obtained from the assays reflect their distinct reaction mechanisms and solvent compatibility: DPPH, operating in organic solvents, is more responsive to lipophilic antioxidants; FRAP, which functions under acidic aqueous conditions (pH 3.6), favors electron-donating hydrophilic compounds; while ABTS, with its broader pH range and solubility, can detect both hydrophilic and lipophilic antioxidants due to its mixed-mode reactivity, i.e., hydrogen atom transfer (HAT) and single electron transfer (SET) [71]. In contrast, IRD-dried samples consistently exhibited the lowest antioxidant activity across all assays: DPPH (14.76 mg TE/g d.m.), ABTS (33.34 mg TE/g d.m.), and FRAP (24.99 mg TE/g d.m.). This pronounced reduction may be explained by the intense thermal energy emitted by infrared lamps, which can promote the oxidation of phenolic compounds (such as phenolic acids, flavonoids, and anthocyanins) during processing [72], which aligns with the lower retention of such compounds observed in IRD samples before digestion in the present study (Table 1 and Table 2).

**Figure 6:**
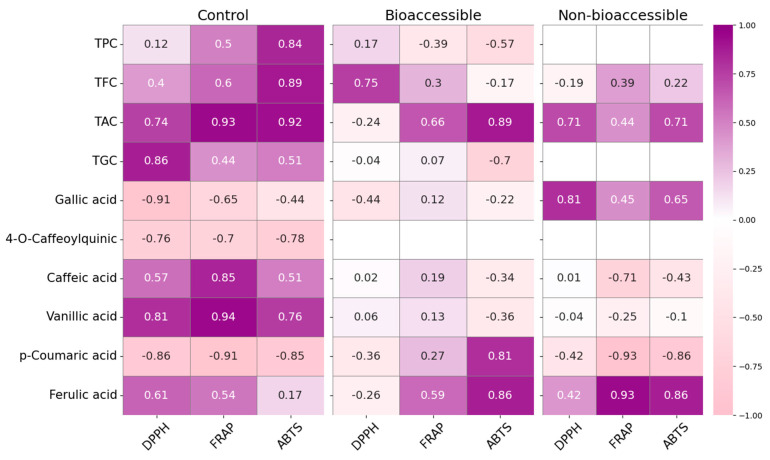
Pearson correlation coefficients between antioxidant activity (DPPH, FRAP, ABTS) and biactive compound variables (TPC, TFC, TAC, TGC, and individual phenolic acids) in red cabbage extracts. Correlations are shown separately for control (non-digested), bioaccessible, and non-bioaccessible fractions. Positive correlations are represented in purple and negative in pink.

A Pearson correlation analysis in the non-digested (control) samples is presented in Figure 6.

Strong positive correlations were observed between TFC and antioxidant activity, particularly with assays ABTS (r = 0.89) and FRAP (r = 0.60), as well as between TAC and both ABTS (r = 0.92) and FRAP (r = 0.93), suggesting that flavonoids and anthocyanins were the main contributors to antioxidant capacity in undigested samples. Caffeic and vanillic acids also correlated strongly with FRAP (r = 0.85 and 0.94, respectively) and TPC with ABTS (r = 0.84), confirming their relevance as potent electron donors. Meanwhile, DPPH values were only strongly correlated with vanillic acid (r = 0.81), suggesting that the antioxidant response in this assay may be driven by specific low-molecular-weight phenolics. This could be partially explained by the sterically hindered structure of the DPPH radical, which limits its accessibility to bulkier or highly glycosylated phenolic compounds, thereby reducing overall reactivity with complex phenolic mixtures [71].

When comparing the radical scavenging assays DPPH and ABTS after in vitro digestion, we found that CD-, VD-, and IRD-dried samples exhibited significant increases in DPPH activity (171.3% in CD, 120.6% in VD, and 136.8% in IRD). This suggests the release of hydrogen-donating compounds that were previously bound within the matrix [73]. In contrast, LTVD and FD samples, which initially displayed high DPPH values, showed a slight decrease after digestion (87.2% and 91.5%, respectively), possibly due to the preservation of oxidative enzymes during low-temperature drying. These enzymes may have remained active during digestion and contributed to the degradation of certain phenolic compounds [18]. The ABTS assay, on the other hand, revealed more pronounced increases in FD (204%) and VD (161%) samples, suggesting that digestion improved the availability of compounds with high electron-donating potential and mixed polarity [74]. The differences observed between the DPPH and ABTS assays after digestion can be explained by the potential release and reactivity of polyphenols from the food matrix under gastrointestinal conditions. During digestion, particularly in the gastric phase, phenolic compounds can form stable complexes with macromolecules such as proteins, carbohydrates, lipids, or even digestive enzymes (e.g., pepsin, trypsin) [49,74]. These complexes remain stable in the acidic environment of the stomach, limiting the immediate availability of free polyphenols. However, upon transition to the intestinal phase, where the pH shifts to neutral or slightly alkaline, these complexes tend to dissociate, resulting in the release of previously bound phenolic compounds [46,73,75]. Once free, these polyphenols can donate hydrogen atoms from their hydroxyl groups to neutralize free radicals, forming stable phenoxyl radicals [76], or undergo electron transfer reactions depending on their structure [74]. This selective release and activation of phenolics contribute to the increased antioxidant capacity observed after digestion. In particular, the balance between two mechanisms, HAT and SET, can determine how effectively each polyphenol interacts with different radicals. For instance, DPPH, which primarily relies on HAT, is more responsive to compounds with readily available hydroxyl groups, whereas ABTS, with its mixed-mode reactivity (HAT/SET), can interact with a broader range of polyphenols, including those with higher redox potential [74]. However, the underlying reaction mechanisms cannot be strictly classified as HAT or SET, since both can occur simultaneously in varying proportions depending on the concentration and structure of the antioxidant, as well as the solvent and pH [71]. Typically, electron transfer is faster than hydrogen transfer, which may explain why ABTS responds more quickly and intensely to a wider range of compounds, as shown by the Pearson correlation analysis (Figure 6). In the bioaccessible fraction, ABTS activity correlated strongly with p-coumaric acid (r = 0.81) and ferulic acid (r = 0.86), indicating that these phenolic acids play a major role in the enhanced antioxidant capacity detected after digestion. Their chemical structure, particularly their conjugated aromatic systems and hydroxyl groups, facilitates electron transfer, supporting ABTS scavenging activity [76]. Meanwhile, DPPH activity showed weak or negligible correlations with all individual phenolics in the bioaccessible fraction, suggesting that the DPPH radical is sterically hindered and reacts more slowly with phenolic compounds released during digestion compared to ABTS, which may underestimate the activity of certain antioxidants [71].

Regarding the FRAP assay, based on a SET mechanism, it measures the ability of antioxidants to reduce ferric ions (Fe^3^⁺) to ferrous ions (Fe^2^⁺) under acidic conditions (pH 3.6). This reaction is favored in low pH environments where the redox potential increases and the ionization potential of antioxidants decreases, facilitating electron transfer [77]. In this study, unlike DPPH and ABTS, FRAP values (associated with hydrophilic electron-donating capacity) were significantly reduced after digestion across all drying methods. The bioaccessible fraction retained only 67.2% to 86.1% of the initial antioxidant content, with the most pronounced reduction observed in IRD-dried samples, which showed the lowest reducing power in all fractions. These reductions are likely due to the degradation or transformation of pH-sensitive compounds, such as flavonols, under acidic gastric conditions. Flavonol modifications, like reduced hydroxylation in the A and/or B rings or hydrogenation of the C-ring double bond or glycosylation at the 3-OH position, can hinder their ability to donate electrons, thereby lowering their FRAP response [43]. However, Pearson correlation analysis showed that TAC (r = 0.66) and TFC (r = 0.30) still correlated positively with FRAP in the bioaccessible fraction, suggesting that anthocyanins and, to a lesser extent, flavonoids that survive digestion or are newly released from the matrix continue to contribute to the ferric-reducing power. Moreover, ferulic acid also showed a moderate positive correlation with FRAP (r = 0.59), indicating its relevant role in maintaining antioxidant capacity after gastrointestinal processing. These results confirm that, although total antioxidant levels decline post-digestion, a subset of stable or accessible phenolic compounds still exerts measurable activity through electron transfer mechanisms.

In all assays, residual antioxidant capacity was detected in the non-bioaccessible fractions, which may be attributed to phenolic compounds that remain bound to the food matrix and are not released during digestion. This interpretation is supported by Pearson correlation analysis, which showed strong positive correlations between antioxidant activity and specific phenolics in the non-bioaccessible fraction. Notably, ferulic acid exhibited strong correlations with FRAP and ABTS values (r = 0.94 and 0.81, respectively), while gallic acid showed a strong correlation with DPPH values (r = 0.81). These results suggest that, despite limited release, structurally stable or matrix-bound antioxidants may still exert measurable activity and could become available in later digestive stages or through microbial fermentation in the colon [20].

## 4. Conclusions

In summary, the microstructural modifications of red cabbage caused by different drying methods affect the bioaccessibility of its bioactive compounds and its antioxidant capacity after digestion. The data acquired in this study demonstrated that while certain compounds, such as anthocyanins, vanillic acid, and 4-O-caffeoylquinic acid in dried red cabbage, degrade during in vitro gastrointestinal digestion, others—particularly glucosinolates, flavonoids, ferulic, p-coumaric, and gallic acids—become more bioaccessible. This phenomenon highlights the pivotal role of different drying methods. Both FD and LTVD contributed to better preservation of the porous microstructure in dried red cabbage, which likely enhanced the release of phenolic compounds from the matrix. This structural integrity may have favored the extractability and/or bioaccessibility of these compounds, thereby supporting their antioxidant potential to some extent. However, it is important to note that these conclusions are based solely on in vitro digestion models, which do not fully replicate the complexity of human digestion and absorption. Therefore, further in vivo studies or advanced in vitro colonic digestion experiments are necessary to confirm these findings, particularly to elucidate the role of myrosinase in the degradation of glucosinolates into isothiocyanates and to assess their true bioaccessibility.

Finally, while FD and LTVD showed promising results in preserving functional quality, their implementation at an industrial scale may face challenges due to high energy consumption, longer processing times, and greater equipment investment. These factors may limit their practicality in large-scale food production. Future research should explore process optimization or hybrid technologies that can achieve a balance between product quality and industrial feasibility.

## Figures and Tables

**Table 1:** Change in the total polyphenol content (TPC), total flavonoid content (TFC), total anthocyanin content (TAC), and total glucosinolates content (TGC) of dried red cabbage with different methods during in vitro gastrointestinal digestion.

Parameter; Extract	Drying Methods				
	CD	VD	IRD	LTVD	FD
TPC (mg GAE/g d.m.); Control	12.64 ± 1.07 ^aB^	11.22 ± 0.01 ^bA^	10.53 ± 0.17 ^bB^	11.29 ± 0.26 ^bB^	11.39 ± 0.83 ^abB^
TPC (mg GAE/g d.m.); Bioaccessible	21.92 ± 1.72 ^bA^	13.09 ± 2.38 ^cA^	31.14 ± 3.28 ^aA^	21.03 ± 0.65 ^bA^	15.73 ± 1.83 ^cA^
TPC (mg GAE/g d.m.); Non-bioaccessible	n.d.	n.d.	n.d.	n.d.	n.d.
TFC (mg CAE/g d.m.); Control	4.49 ± 0.18 ^aB^	3.78 ± 0.09 ^bA^	3.84 ± 0.04 ^abB^	4.18 ± 0.18 ^aA^	4.20 ± 0.22 ^aA^
TFC (mg CAE/g d.m.); Bioaccessible	5.09 ± 0.15 ^aA^	4.02 ± 0.35 ^cA^	4.74 ± 0.46 ^abA^	4.24 ± 0.34 ^bcA^	4.45 ± 0.07 ^bcA^
TFC (mg CAE/g d.m.); Non-bioaccessible	0.49 ± 0.06 ^aC^	0.38 ± 0.05 ^abB^	0.37 ± 0.03 ^bC^	0.39 ± 0.05 ^abB^	0.42 ± 0.12 ^abB^
TAC (mg Cya3glu/g d.m.); Control	5.03 ± 0.70 ^abA^	4.30 ± 0.38 ^bA^	2.62 ± 0.20 ^cA^	5.17 ± 1.26 ^aA^	5.65 ± 0.62 ^aA^
TAC (mg Cya3glu/g d.m.); Bioaccessible	3.84 ± 0.23 ^bcB^	4.01 ± 0.42 ^bA^	2.94 ± 0.31 ^dA^	3.57 ± 0.24 ^cB^	5.72 ± 0.48 ^aA^
TAC (mg Cya3glu/g d.m.); Non-bioaccessible	0.76 ± 0.07 ^abC^	0.49 ± 0.04 ^cB^	0.47 ± 0.05 ^cB^	0.86 ± 0.09 ^aC^	0.67 ± 0.07 ^bB^
TGC (µmol SE/g d.m.); Control	85.94 ± 4.25 ^abB^	87.65 ± 1.78 ^abB^	78.53 ± 2.44 ^bB^	94.78 ± 9.70 ^aB^	86.00 ± 2.49 ^abB^
TGC (µmol SE/g d.m.); Bioaccessible	109.50 ± 4.95 ^bcA^	107.91 ± 9.44 ^bA^	106.81 ± 11.92 ^bA^	146.59 ± 13.41 ^aA^	101.49 ± 4.79 ^cA^
TGC (µmol SE/g d.m.); Non-bioaccessible	n.d.	n.d.	n.d.	n.d.	n.d.

CD: convective drying; VD: vacuum drying; IRD: infrared drying; LTVD: low-temperature vacuum drying; FD: freeze-drying. Different lowercase letters in the same row indicate significant differences (*p* < 0.05) among drying techniques. Different uppercase letters in the same column indicate significant differences (*p* < 0.05) among digested samples. Values are expressed as mean ± standard deviation. Standard deviation was calculated on three replicates. Total phenolic content (TPC); Total flavonoid content (TFC); Total anthocyanin content (TAC); Total glucosinolate content (TGC); Gallic acid equivalents (GAE); Cathequin equivalents (CAE); cyanidin-3-glucoside (cya3glu); Sinigrin equivalents (SE). n.d.: not detected.

**Table 2:** Changes in the individual phenolic compounds of dried red cabbage using different methods during in vitro gastrointestinal digestion.

Phenolic Acids (mg/g d.m.)		Drying Methods				
		CD	VD	IRD	LTVD	FD
Gallic acid	Control	0.18 ± 0.00 ^aA^	0.18 ± 0.00 ^aB^	0.18 ± 0.01 ^aA^	n.d.	n.d.
	Bioaccessible	0.11 ± 0.01 ^cB^	0.26 ± 0.01 ^bA^	0.12 ± 0.00 ^cA^	0.47 ± 0.09 ^aA^	0.24 ± 0.01 ^bA^
	Non-bioaccessible	0.02 ± 0.00 ^bC^	0.02 ± 0.00 ^bC^	0.03 ± 0.00 ^bB^	0.04 ± 0.00 ^aB^	0.01 ± 0.00 ^cB^
4-O-Caffeoylquinic	Control	0.10 ± 0.00 ^bc^	0.10 ± 0.01 ^b^	0.15 ± 0.01 ^a^	0.08 ± 0.00 ^c^	0.10 ± 0.01 ^bc^
	Bioaccessible	n.d.	n.d.	n.d.	n.d.	n.d.
	Non-bioaccessible	n.d.	n.d.	n.d.	n.d.	n.d.
Caffeic acid	Control	0.06 ± 0.00 ^cA^	0.06 ± 0.00 ^cB^	0.06 ± 0.00 ^dA^	0.09 ± 0.00 ^bA^	0.18 ± 0.00 ^aA^
	Bioaccessible	0.07 ± 0.00 ^bcA^	0.08 ± 0.01 ^abA^	0.06 ± 0.00 ^dA^	0.09 ± 0.01 ^aA^	0.07 ± 0.01 ^cdB^
	Non-bioaccessible	0.03 ± 0.00 ^cB^	0.05 ± 0.00 ^bC^	0.04 ± 0.00 ^cB^	0.05 ± 0.01 ^bB^	0.07 ± 0.01 ^aB^
Vanillic acid	Control	0.40 ± 0.02 ^cA^	0.29 ± 0.00 ^dA^	0.09 ± 0.00 ^eA^	0.60 ± 0.01 ^bA^	0.87 ± 0.06 ^aA^
	Bioaccessible	0.19 ± 0.01 ^bB^	0.25 ± 0.04 ^aA^	0.11 ± 0.01 ^cA^	0.27 ± 0.04 ^aB^	0.15 ± 0.02 ^bcB^
	Non-bioaccessible	0.01 ± 0.00 ^dC^	0.01 ± 0.00 ^cB^	n.d.	0.01 ± 0.00 ^bC^	0.02 ± 0.00 ^aC^
p-Coumaric acid	Control	0.08 ± 0.00 ^cB^	0.10 ± 0.00 ^bB^	0.12 ± 0.00 ^aB^	0.06 ± 0.00 ^dB^	0.05 ± 0.00 ^dB^
	Bioaccessible	0.44 ± 0.02 ^bA^	0.43 ± 0.02 ^bA^	0.49 ± 0.03 ^bA^	0.41 ± 0.06 ^bA^	0.58 ± 0.07 ^aA^
	Non-bioaccessible	0.07 ± 0.00 ^aB^	0.07 ± 0.01 ^aB^	0.08 ± 0.00 ^aB^	0.07 ± 0.01 ^aB^	0.08 ± 0.01 ^aB^
Ferulic acid	Control	0.02 ± 0.00 ^bC^	0.03 ± 0.00 ^aB^	0.02 ± 0.01 ^bB^	0.03 ± 0.00 ^aC^	0.03 ± 0.01 ^aB^
	Bioaccessible	0.37 ± 0.02 ^bA^	0.35 ± 0.00 ^bA^	0.36 ± 0.03 ^bA^	0.35 ± 0.01 ^bA^	0.45 ± 0.01 ^aA^
	Non-bioaccessible	0.06 ± 0.00 ^aB^	0.06 ± 0.01 ^aB^	0.05 ± 0.00 ^aB^	0.06 ± 0.01 ^aB^	0.05 ± 0.00 ^aB^
Total	Control	0.84 ± 0.02	0.77 ± 0.01	0.62 ± 0.04	0.86 ± 0.01	1.24 ± 0.09
	Bioaccessible	1.19 ± 0.07	1.38 ± 0.08	1.14 ± 0.07	1.59 ± 0.20	1.49 ± 0.11
	Non-bioaccessible	0.19 ± 0.01	0.21 ± 0.02	0.20 ± 0.01	0.24 ± 0.03	0.23 ± 0.02

CD: convective drying; VD: vacuum drying; IRD: infrared drying; LTVD: low-temperature vacuum drying; FD: freeze-drying. Different lowercase letters in the same row indicate significant differences (*p* < 0.05) among drying methods. Different uppercase letters in the same column indicate significant differences (*p* < 0.05) among digested samples. Values are expressed as mean ± standard deviation. Standard deviation was calculated on three replicates. n.d.: not detected.

## Data Availability

The original contributions presented in the study are included in the article, further inquiries can be directed to the corresponding author.
